# Ontogenetic Change in Performance: Do Innovations Constrain Performance in Gobiid Fishes?

**DOI:** 10.1093/iob/obag044

**Published:** 2026-08-04

**Authors:** H L Schoenfuss, K M Diamond, R Lagarde, R W Blob

**Affiliations:** Aquatic Toxicology Laboratory, St Cloud State University, St. Cloud, MN 56301, USA; Dept of Biology, Rhodes College, Memphis, TN 38112, USA; Université de Perpignan Via Domitia - CNRS, Centre de Formation et de Recherche sur les Environnements Méditerranéens, UMR 5110, F 66860 Perpignan, France; Dept of Biological Sciences, Clemson University, Clemson, SC 29634, USA

## Abstract

Recent innovations in a species may expand opportunities to obtain new resources or previously inaccessible habitats. Over time, the novel behavior may diversify in response to an adaptive landscape or become canalized by convergent selective pressures into a narrowed performance window. Selective pressures also change across life stages, which may further enhance diversification or constrain performance. To test these alternative hypotheses, we compared ontogenetic changes in the performance of a novel locomotor behavior—waterfall-climbing—across five amphidromous species of gobiid fishes from the Pacific, Caribbean, and Indian Oceans. Two species climb waterfalls using an inching motion, a derived functional innovation that alternates movements of pelvic and oral suckers. The remaining species climb waterfalls using short “powerbursts” of swimming, followed by long rest periods in which the pelvic sucker is attached to the waterfall substrate. Among adults, net climbing speed (including rest) was similar across the three powerburst species and one inching species, but a derived, faster speed was observed in one inching taxon, suggesting greater functional diversity in the younger innovation. However, kinematic data indicate that similar performance levels can be achieved through multiple functional pathways. Moreover, patterns of functional diversity across species observed in juveniles do not track neatly into adulthood, highlighting the range of potential influences that can shape the trajectory of ontogenetic change in function.

## Introduction

The evolution of novel behaviors can provide organisms with opportunities to access previously unavailable habitats and resources (Simpson 1944; Wainwright and Price 2016). With time, the behaviors may diversify in response to selective pressures imposed by the adaptive landscape (Jablonski and Bottjer 1990; Wainwright and Price 2016) or through the accumulation of changes via chance events (Lynch and Hill 1986). Alternatively, selective pressures over time might constrain how a novel behavior is implemented, resulting in a narrow performance window (Westneat and Wainwright 1989; Galpern 2000; Hansen and Houle 2004). Additionally, the evolution of a novel function in response to current functional needs may constrain the range of structural adaptations. Discerning factors that determine whether new functional traits diversify or become constrained through the course of evolution could help to inform predictive understanding of species responses to introduced stressors or environmental change (Wainwright and Price 2016).

A complicating factor in evaluating such possibilities for many species is that they are exposed to changing conditions throughout their life history. Simply through growth in body size, many organisms may experience changes in their physical environment and biological interactions (Carrier 1996; Toro et al. 2003; Herrel and Gibb 2006; Blob et al. 2007). With size increases, the diversity of how groups of related species perform functions could become constrained (Hansen and Houle 2004; Wainwright and Price 2016). However, across these groups, the diversity of functional performance in adults might also reflect patterns established early in life and correspond to the diversity observed in juveniles (Blob et al. 2008b).

**Fig. 1:**
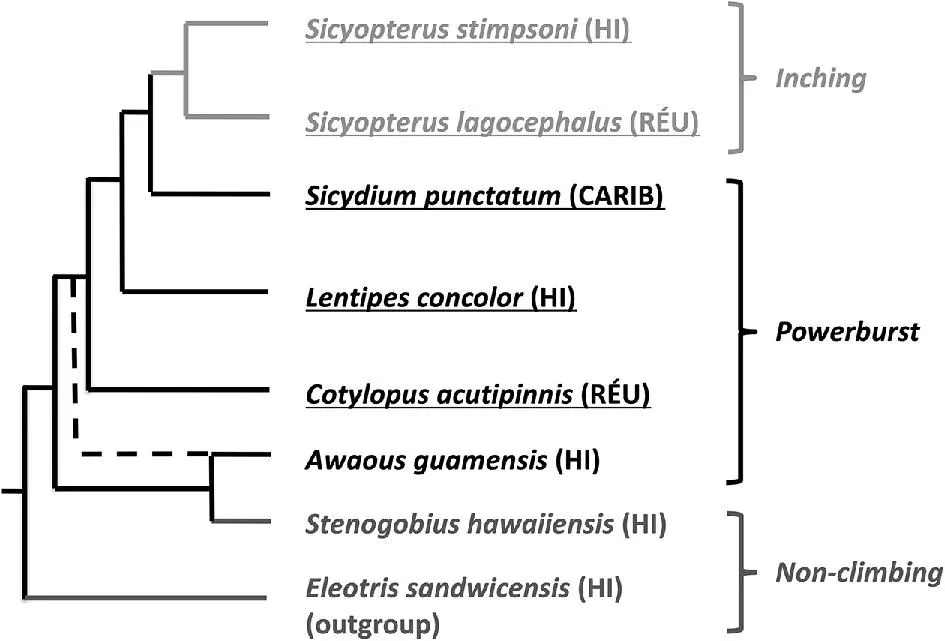
Simplified phylogeny of Sicydiine gobies and outgroups, illustrating the evolutionary distribution of the powerburst and inching styles of climbing of fishes found in Hawaii (HI), La Réunion (RÉU), and the Caribbean (CARIB). Underlined species are evaluated in the current study. Phylogeny after Thacker (2003), Keith et al. (2011), and Taillebois et al. (2014); diagram modified from Blob et al. (2019).

To test these alternative possibilities, we evaluated the functional performance of adults and juveniles from multiple amphidromous gobioid species in the Pacific, Caribbean, and Indian Oceans that use distinct climbing styles to scale waterfalls during their juvenile upstream migration and retain this ability into adulthood. Migratory species experience bouts of intense selective pressure due to the demands of migration across different environments (Jansen et al. 2007; Blob et al. 2010; Schoenfuss et al. 2011; Kawano et al. 2013; Oppel et al. 2015). Often, these migrations are limited to a brief period of ontogeny. However, the consequences of selective pressures may constrain function throughout the rest of these individuals’ lives. Moreover, our comparisons included species with two distinct climbing styles (Fig. 1), allowing us to assess whether older functional innovations might be less conserved than more recent ones due to the accumulation of variation over time (Wainwright and Price 2016).

Many amphidromous stream gobies face intense selective pressures during their juvenile migration from near-shore oceans to adult stream habitats (Blob et al. 2008a; Blob et al. 2010; Diamond et al. 2019; Schneider et al. 2021). During their upstream migration, waterfalls pose formidable barriers that fish must scale to reach adult habitats (Kawano et al. 2013). However, two distinct climbing styles have been recognized among these fishes: “powerburst climbing” in which fish alternate short bursts of axial undulation with longer rest periods when the animal attaches to the waterfall surface using a sucker formed from fused pelvic fins; and “inching,” which consists of alternating attachment to the waterfall surface by the pelvic sucker and a novel oral sucker (Schoenfuss and Blob 2003). Waterfall climbing by inching has only been observed in the genus *Sicyopterus*, which is phylogenetically nested within several powerburst outgroups. Phylogenetic analysis has placed the origin of the Sicydiinae at the Miocene, around 12–6 mya (Taillebois et al. 2014), with the sister groups of *Sicyopterus* and *Sicydium* emerging more recently, but no later than 4–3 mya ago based on the dispersal hypothesis through the Panamanian isthmus (Taillebois et al. 2014). Thus, the inching behavior observed in *Sicyopterus* seems to be a substantially more recently evolved mode of climbing (Cullen et al. 2013), although the exact length of time since its divergence has not been established. Powerburst climbers may have accumulated more functional variation in their climbing performance than inchers due to their earlier evolutionary origin. Moreover, inching requires alternate attachments of a pelvic sucking disc, an ancestral, diagnostic feature of gobies used for attachment on many substrates, and an oral sucking disc, a novel derivation in which the mouth is co-opted as a secondary locomotor organ (Cullen et al. 2013). Since inching climbers are algal grazers, it is conceivable that functional variation in their climbing style is constrained not only by more recent evolutionary derivation but also by using the mouth as a feeding organ as well as locomotor structure.

Previous studies of migrating juveniles have identified distinct differences in the kinematics, physiology, and performance of the two climbing styles (reviewed in Blob et al. 2023). Powerburst climbing is powered primarily by contraction of anaerobic axial musculature, whereas inching climbing involves more substantial contributions from aerobic muscle (Cediel et al. 2008a; Schoenfuss et al. 2013; Lagarde et al. 2020). As a result, powerburst climbers reach greater velocities during climbing but spend more time resting than the slow but steady inching climbers. Regardless of climbing style, all waterfall climbing gobioid fishes need to generate sufficient suction forces to maintain station while attached to the surface of the waterfall by their pelvic sucker or, in inching climbers, also by the oral disk (Maie et al. 2012). Adult gobioid species generally retain climbing ability, although climbing performance may diminish with increasing size in some species, and some species cease to climb at their largest sizes (Blob et al. 2007; Lagarde et al. 2021). Comparative studies of juvenile powerburst and inching climbing species (Blob et al. 2019) indeed revealed greater variability in climbing performance among juvenile powerburst climbers (net climbing speed) than among juvenile inching climbers, though closer examination revealed hidden components of functional diversity in inchers, which achieved similar climbing speeds via different mechanisms (i.e., fast movements interspersed with long rest periods, versus slower movements interspersed with less rest; Blob et al. 2019).

In the current study, we collected data from adults of two further species of waterfall-climbing gobies, *Cotylopus acutipinnis* (a powerburst climber) and *Sicyopterus lagocephalus* (an inching climber), for which climbing performance and kinematics of adults have not been previously evaluated. By comparing climbing kinematics and performance of juveniles and adults for four powerburst climbers from three volcanic islands (Hawai’i—Pacific; Dominica—Caribbean; La Réunion—Indian Ocean) with two species of inching climbers from islands in two different ocean basins (Hawai’i; La Réunion), we can address two questions: (i) is the ancestral, powerburst climbing style less conserved in kinematics and performance than more recent locomotor innovations like inching (Blob et al. 2019); and (ii) are the performance constraints related to the different climbing styles of juveniles retained in adult fishes.

## Materials and methods

### Fish collections

Adult *Lentipes concolor* and *Sicyopterus stimpsoni* on the Island of Hawai’i (Hakalau stream), and *Sicydium punctatum* in Dominica (Check Hall River) were collected with “opae nets” (bowl-shaped mesh nets) while snorkeling (Blob et al. 2007; Schoenfuss et al. 2011). Juveniles of these species were captured with small dipnets near waterfalls (Blob et al. 2019). Because the turbidity of rivers on La Réunion during our field period prevented capturing fish while snorkeling, juvenile and adult *Sicyopterus lagocephalus* and *Cotylopus acutipinnis* were collected by electroshocking in the St. Etienne and Marsouins rivers on La Réunion in the Indian Ocean (Lagarde et al. 2020). In all instances, fish were transported in insulated coolers filled with aerated ambient stream water to a nearby research station within 5 hrs of capture and maintained for up to 72 hrs in aerated containers filled with fresh water that was exchanged daily. Fish capture, housing, and experimentation were approved by St. Cloud State University Institutional Animal Care and Use Committee (IACUC; protocols 12–07). Sampling in La Réunion was performed under permit No. 15–002/DEAL/SEB/UPEMA issued by the Direction de l’Environnement de l’Amenagement et du Logement de La Réunion. No mortality was associated with the experimental procedures, and, except for voucher specimens, the experimental fish were returned to their native stream after experimentation.

### Recordings of climbing performance and kinematics

Across both ontogenetic stages, all species, and all field sites, we attempted to replicate climbing structures using the methodology detailed in Blob et al. (2006, 2007). Briefly, for measurements of climbing performance over a set distance (20 cm), a small basin containing a rock at its center was filled to a depth of 8 cm with ambient-temperature stream water and placed below a climbing chute angled at 70°. The plastic chute was coated in fine sand and contained a scale bar along its edge. A camera (30Hz Sony DTV 1020 mini-DV camera) was placed perpendicular to the chute surface and recorded the dorsal surface of the fish for the first 20 cm of climbing. Ambient-temperature stream water was directed across the chute at approximately 200 mL/min. Juvenile or adult fish were placed into the basin and readily climbed these chutes, often within 1–2 min. Each fish was used in a single trial. To assess climbing performance over the 20 cm chute surface in the field of view, the 30Hz recordings were transferred to a Macintosh computer and analyzed using iMovie software (various versions). Climbing bout duration and length of intervening rest periods were determined. Detailed methodology is described in Blob et al. (2006, 2007).

To assess climbing kinematics, 125 Hz recordings (HiSpec 2 digital high-speed video camera) were collected from a ventral view through the Plexiglas climbing surface and saved as AVI files. A higher frame rate was necessary for climbing kinematics than for performance analysis due to the higher velocities achieved by powerburst juveniles during individual locomotor cycles. To avoid biases in kinematic analyses of powerburst and inching climbers, the same 125 Hz frame rate was used for all species and both ontogenetic stages. Anatomical landmarks were digitized frame-by-frame using DLTdv5 for MATLAB (Hedrick 2008). Points at the base and tip of both pectoral fins were marked, along with four points on the head representing the anterior midline of the face and the left and right lateral and posterior margins of the mouth. At least nine additional points along the body midline were also digitized, with the most posterior point at the base of the caudal peduncle.

Custom code in MATLAB (Version 5.0; MathWorks, Inc.; Natick, MA, USA) was used to calculate kinematic variables. To describe the movement of inching climbers during waterfall climbing, three kinematic variables representing anatomical structures engaged in upward movement were quantified in *S. stimpsoni* and *S. lagocephalus*. Front lip displacement describes the incremental anterior (i.e., upwards) movement of the upper lip during climbing. For each frame of a locomotor cycle, the anterior edge of the upper lip at the midline was digitized. Using a custom MATLAB routine, these coordinates enabled calculation of the lip’s instantaneous position throughout the climbing cycle. Given the nature of the inching climbing style, displacement of the anterior lip pauses while the mouth is used as a sucking disc to attach to the climbing substrate. During attachment, the mouth area should enlarge (similar to a suction cup pressed against a surface). To quantify the instantaneous surface area enclosed by the oral sucking disc, we digitized landmarks representing the left and right edges of the mouth, as well as the anterior and posterior edges, and used these coordinates to calculate a simplified mouth area. Although this calculation simplifies the mouth’s shape, it provides a measure of whether the mouth area increases or decreases during the climbing cycle. Lastly, to quantify fin movement in both inching and powerburst species, we determined the angle between vectors formed from the base and tip of each pectoral fin along with 2 points along the midline of the head to serve as a reference for the instantaneous direction of travel. From these vectors, the angle of each fin relative to the direction of travel was calculated throughout the climbing cycle to determine when the fins contribute to thrust generation.

Lastly, pressure differentials generated by the pelvic sucker, a diagnostic feature of gobioid fishes, and the oral sucker, specific to the genus *Sicyopterus*, were measured. This procedure closely followed the method described by Maie et al. (2012). Briefly, a pressure transducer with a data acquisition interface (SensorDAQ, Vernier Software & Technology, Beaverton, OR, USA) was connected via a cannula to a hole in the climbing surface. Pressure differentials were recorded as fish moved over the hole while climbing the chute. High-speed recordings from these trials were used to confirm that each fish moved directly across the hole, as in the trials used for statistical analyses.

### Statistical analyses

Statistical analyses were performed using GraphPad Prism (San Diego, CA, USA) and in the R environment (R Core Team 2021, version 4.1.2). The mean locomotor cycle duration and maximum fin angle for each species were calculated from high-speed video, whereas the mean durations of climbing bouts, climbing velocities, and the % time spent in motion for entire climbing bouts (over the 20 cm distance) were calculated from standard video. Net climbing speed (accounting for rest periods) was calculated by dividing the 20 cm distance by the sum of climbing and rest times. “Climbing-only” climbing speed, based only on the time during which a fish was actively climbing, was also calculated from standard video. In addition, the percentage of time that each individual spent moving was calculated as time in motion divided by the sum of motion and rest times. The assumption of normality for all datasets was tested using the Dagostino-Pearson omnibus test (GraphPad Prism). Because many datasets did not meet the homogeneity assumption, the difference between species was assessed using a Kruskal-Wallis rank test followed by Dunn’s post hoc tests with a Bonferroni correction. Similarly, intraspecific variability in juveniles or adults within each species was compared using a non-parametric Fligner-Killeen test of homogeneity of variance, followed by a pairwise comparison with a Bonferroni correction.

The scaling relationships between body mass and pelvic and oral sucker pressure differential and between durations of climbing bouts, climbing velocities, the % time spent in motion and body length were addressed using model II reduced major axis regressions (RMA) fitted separately for each species and each response variable (Maie et al. 2007, 2012). These analyses included data from our new measurements of adults from Réunion and previously reported data from juveniles of these species (Blob et al. 2019) as well as, where available, previously reported data from adults and juveniles of climbing species from Dominica (Schoenfuss et al 2011) and Hawai’i (Schoenfuss and Blob 2003; Blob et al. 2006, 2007). The body mass and sucker pressure differential were log-transformed to account for the quadratic or cubic increase of areas and volume with size. For all models, an isometric scaling relationship was considered if the regression coefficient did not differ significantly from 0. In contrast, a negative or positive slope was considered as a negative or positive isometric scaling, respectively. The RMA regression was fitted using the lmodel2 R package (Legendre 2018).

## Results

Concurrent with observations of other gobioid species in our previous studies (Blob et al. 2007; Schoenfuss et al. 2011), adult *C. acutipinnis* (n = 15; 4.2 ± 0.98 cm; range 3.0–6.52 cm) and *S. lagocephalus* (n = 41; 6.5 ± 1.8 cm; range: 4.58–14.86 cm) climbed readily when placed into the basin below the artificial waterfall, with climbing usually commencing within the first minute after placement. Adult individuals of *C. acutipinnis* were smaller than the two other powerburst species in the current study, while *S. lagocephalus* individuals were larger than the other inching climber, *S. stimpsoni* (n = 25; 6.4 ± 1.2 cm; range: 4.16–6.07 cm; Table 1).

**Table 1:** Sample size (n), mean values (mean), and significant differences between juveniles or adults of species as signified by letters (a, b, c) in the “sign.” column indicate significant differences between treatments within a row (*P* < 0.05, Kruskal-Wallis with Dunn’s post-test). Standard deviations (st dev) of morphological, kinematic, and performance variables for adult waterfall-climbing stream gobies from Hawai’i, Dominica, and Réunion, and significant differences in intraspecies variability signified by letters (a, b, c) in the “sign*” column (non-parametric Fligner–Killeen test of homogeneity of variance, followed by a pairwise comparison with a Bonferroni correction). Juveniles and adults were analyzed separately (i.e., letters “a, b, c” apply to individual rows). Data for Hawaiian and Dominican gobioid fishes are derived from previously published studies (Schoenfuss and Blob 2003; Blob et al. 2006, 2007; Schoenfuss et al. 2011; Maie et al. 2012). “n/a” not applicable. [BL/s] indicates climbing velocity adjusted to the relative size of each fish (body lengths/second). ^1^Data derived from 30Hz DV recordings; ^2^data derived from high-speed video recordings; ^3^data derived from suction measurements.

		Hawaii										Dominica					Reunion											
		*Lentipes concolor (powerburst)*					*Sicyopterus stimpsoni (inching)*					*Sicydium punctatum (powerburst)*					*Cotylopus acutipinnis (powerburst)*					*Sicyopterus lagocephalus (inching)*						
Ontogenic stage	Variable [unit]	*n*	*mean*	*sign.*	*st dev*	sign*	*n*	*mean*	*sign.*	*st dev*	sign*	*n*	*mean*	*sign.*	*st dev*	sign*	*n*	*mean*	*sign.*	*st dev*	sign*	*n*	*mean*	*sign.*	*st dev*	sign*	Kruskal-Wallis *P*-value	Fligner-Kileen *P*-value
	**Fish length^1^ [cm]**	43	6.1	ac	1.1	n/a	25	5.4	ab	1.2	n/a	15	6.3	ac	0.76	n/a	15	4.2	b	0.98	n/a	41	6.5	c	1.8	n/a	<0.001	0.06
	**Climbing bout duration^1^ [s]**	43	2.7	a	2.6	a	25	4.7	bcd	3.1	bc	15	2.7	ac	0.6	bd	15	2.8	ad	0.8	ad	41	4.5	b	1.7	acd	<0.001	0.001
	**Net climbing speed including rest^1^ [cm/s]**	43	1	ac	1.3	a	25	0.71	a	0.49	a	15	0.22	bd	0.15	ac	15	0.34	bc	0.12	bc	41	0.19	d	0.099	bc	<0.001	<0.001
	**Net climbing speed including rest^1^ [BL/s]**	43	0.068	a	0.053	a	25	0.13	b	0.088	a	15	0.037	a	0.027	ac	15	0.048	a	0.03	b	41	0.056	a	0.026	bc	<0.001	<0.001
**Adults**	**Speed during climbing only^1^ [cm/s]**	43	1.2	a	1.4	a	25	0.99	a	0.47	a	15	0.69	ac	0.46	ac	15	1.2	bc	0.27	ac	41	0.79	a	0.42	bc	<0.001	<0.001
	**Speed during climbing only^1^ [BL/s]**	43	0.22	a	0.21	n/a	25	0.17	ab	0.11	n/a	15	0.12	b	0.09	n/a	15	0.3	c	0.079	n/a	41	0.13	b	0.06	n/a	<0.001	0.10
	**Percent time in motion^1^ [%]**	43	37	ad	19	ab	25	67	b	16	ab	15	33	acd	9.7	ab	15	17	c	13	a	41	45	d	15	ab	<0.001	0.021
	**Maximum fin angle^2^ [°]**	32	107	ac	29	a	23	39	b	12	b	50	121	c	17	bc	34	85	a	31	ac	52	24	b	11	b	<0.001	<0.001
	**Cycle duration^2^ [s]**	32	0.23	a	0.049	n/a	23	0.25	a	0.025	n/a	50	0.17	b	0.032	n/a	34	0.12	c	0.027	n/a	52	0.25	a	0.037	n/a	<0.001	0.12
	**Pelvic suction^3^ [kPa]**	9	1.3	n/a	0.4	n/a	15	1.5	n/a	0.97	n/a	12	1.3	n/a	0.61	n/a	21	0.83	n/a	0.81	n/a	16	0.63	n/a	0.48	n/a	0.07	0.17
	**Fish length^1^ [cm]**	17	1.4	a	0	a	16	2.2	bc	0	a	39	1.9	bd	0	a	11	1.6	bd	0.5	b	62	2.5	bc	0.4	b	<0.001	<0.001
	**Climbing bout duration^1^ [s]**	17	1.97	a	0.68	a	16	6.13	b	2.81	bc	39	2.51	a	1.85	ac	11	2.49	ac	0.52	ac	62	3.34	c	1.51	a	<0.001	<0.001
	**Net climbing speed including rest^1^ [cm/s]**	17	0.21	a	0.09	ab	16	0.21	a	0.06	ab	39	0.21	a	0.13	a	11	0.09	b	0.03	ab	62	0.29	a	0.11	a	<0.001	0.04
	**Net climbing speed including rest^1^ [BL/s]**	17	0.15	a	0.06	n/a	16	0.09	ab	0.03	n/a	39	0.11	ab	0.07	n/a	11	0.06	b	0.02	n/a	62	0.11	a	0.05	n/a	<0.001	0.06
	**Speed during climbing only^1^ [cm/s]**	17	0.94	a	0.14	a	16	0.34	bc	0.07	a	39	1.02	a	0.47	b	11	0.73	a	0.25	a	62	0.62	c	0.12	a	<0.001	<0.001
**Juveniles**	**Speed during climbing only^1^ [BL/s]**	17	0.67	a	0.10	a	16	0.15	b	0.03	bc	39	0.54	a	0.25	a	11	0.50	a	0.23	a	62	0.25	c	0.07	ac	<0.001	<0.001
	**Percent time in motion^1^ [%]**	17	22.25	a	10.8	ab	16	22.25	b	10.80	ab	39	21.55	a	11.57	a	11	13.53	a	5.42	ab	62	47.23	b	18.42	a	<0.001	<0.001
	**Maximum fin angle^2^ [°]**	7	121.1	a	11.9	n/a	n/a	n/a	n/a	n/a	n/a	24	98.4	b	15.6	n/a	87	116.9	a	12.8	n/a	n/a	n/a	n/a	n/a	n/a	<0.001	0.39
	**Cycle duration^2^ [s]**	7	0.05	a	0.02	n/a	n/a	n/a	n/a	n/a	n/a	24	0.28	b	0.04	n/a	87	0.22	c	0.03	n/a	n/a	n/a	n/a	n/a	n/a	<0.001	0.15

The duration of climbing bouts matched closely among adults from the three powerburst climbing species but was significantly shorter than in the two inching species. Compared with the two Hawaiian species, adults from the three species from Dominica and Réunion had net climbing speeds (including motion and rest) that were slower (Table 1). Thus, within each of these two groups of species, powerburst and inching climbers achieved similar net performance. As noted in comparisons of juvenile performance (Blob et al. 2019), in adult gobies (Table 1) similar performance between powerburst and inching climbers was achieved through two distinct pathways, with powerburst climbers faster while moving (“Speed during climbing only”) but climbing for a shorter portion of time (“% time in motion”) when compared to inching climbers (Table 1).

**Fig. 2:**
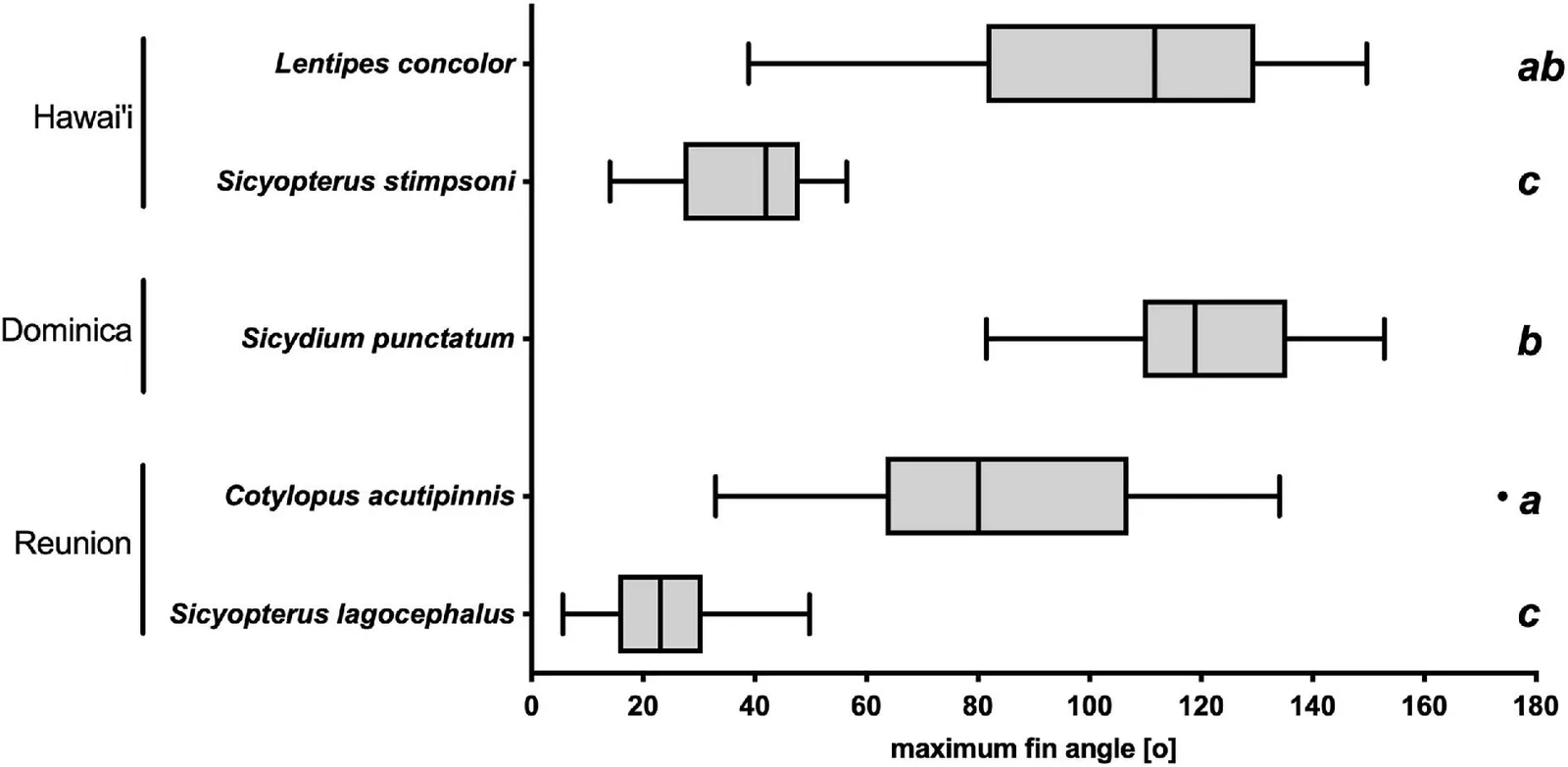
Comparison of maximum fin angles during climbing cycles for adults of five goby species. A box-and-whisker plot shows the data range, the 25^th^ and 75^th^ percentiles, and the median. The dot on the right-hand side of *C. acutipinnis* indicates an outlier (more than 2 standard deviations removed from the mean). Blue shading indicates powerburst species, orange shading indicates inching species. Different lowercase letters indicate significant differences among groups according to Dunn’s post-hoc test following a Kruskal-Wallis test (*P* < 0.05).

**Fig. 3:**
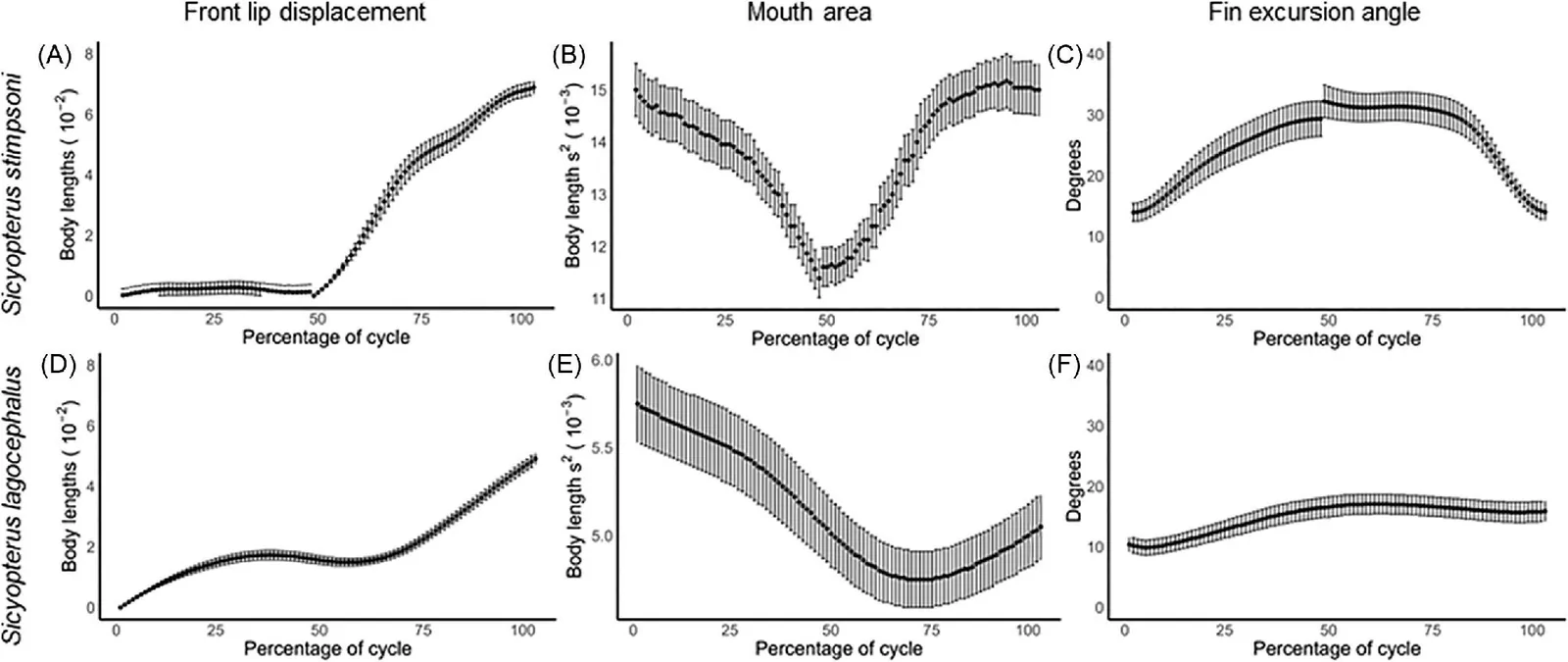
Mean profiles of kinematic variables for *Sicyopterus stimpsoni* (A–C: data replotted from Blob et al. 2007) and *S. lagocephalus* (D–F). Variables include the cumulative displacement of the front lip since the start of the cycle (A, D) and the instantaneous mouth area (i.e., oral sucker: B, E), both normalized by body length. Fin excursion angles for both species are included in C and F. All data are normalized for a single movement cycle.

Climbing kinematics for adult gobies from Réunion corroborated observations made for powerburst and inching climbers from Hawai’i and Dominica (Schoenfuss and Blob 2003; Schoenfuss et al. 2011). Maximum fin angles were three to four times greater in powerburst climbers than in inching climbers, indicating greater excursions that confirm the propulsive significance of fin movement for powerburst climbers (Table 1; Fig. 2). Even though some performance differences were observed among the powerburst climbers, their climbing kinematics revealed comparable maximum fin angles (Fig. 2). For inching climbers, the kinematics of the mouth area, as well as oral sucker attachment, were similar for adults of *S. stimpsoni* on Hawai’i and *S. lagocephalus* on Réunion, though *S. stimpsoni* had slightly higher values for mouth area on average for a given point within the climbing cycle (Fig. 3).

Fligner–Killeen tests for homogeneity of variance did not show clear differences in the variability of performance variables between powerburst and inching species for either juveniles or adults (Table 1). Several variables showed no significant differences in variance across our sample species (for adults: body length, normalized speed during climbing, cycle duration, and pelvic suction; for juveniles, body length, normalized net speed, maximum fin angle, and cycle duration). In addition, although other variables showed significant differences in variance across species, post-hoc tests for all variables failed to show distinct variance groupings between powerburst and inching species (Table 1).

Regressions that tested for allometric patterns in performance variables for each species from juvenile to adult size also failed to show clear evidence for greater variability among powerburst species (Table 2). Among the four performance variables compared across a full range of body size from juvenile to adult (bout duration, net climbing speed, climbing-only speed, and % time in motion), each species seemed to show a distinctive pattern of allometric change. For example, bout duration showed a significant allometric pattern in only one of the five species (negative in inching *S. stimpsoni*), whereas net climbing speed had a significant positive allometry in three out of five species (two powerburst and one inching, with the powerburst species not overlapping in slope). In addition, speed during climbing only showed significant allometric slopes in four out of five species: two powerburst (*C. acutipinnis* having positive allometry and *S. punctatum* having a negative allometry) and the two inching species (*S. stimpsoni* and *S. lagocephalus* with positive but non-overlapping slopes). Moreover, % time in motion showed two powerburst species (*L. concolor* and *S. punctatum*) with significant positive allometry but overlapping slopes and low R^2^ values. If, for example, both inching species had shown similar, overlapping slopes for several variables, it might have indicated that the more recently evolved style of climbing was less variable; however, our scaling analyses did not indicate such patterns.

**Table 2:** Slope and intercept coefficients from RMA regressions (±95% confidence intervals) of climbing performance on size in waterfall-climbing stream gobies from Hawai’i, Dominica, and Réunion. Scaling pattern is indicated as isometric (0), negatively allometric (**-**), or positively allometric (+) compared to the expected slope of 0 for isometry.

Island	species	n	X	Y	r²	*P*-value	Intersept (95% confidence interval)	Slope (95% confidence interval)	Expected RMA by isometry	Allometry
	*Lentipes concolor*	60	Fish length (cm)	Bout duration (s)	0.01	0.46	—	—	0	0
				**Net climbing speed (cm/s)**	**0.15**	**0.002**	**−1.51 (−3.66; −0.38)**	**0.48 (0.24; 0.93)**	**0**	**+**
				Climbing only speed (cm/s)	0.02	0.26	—	—	0	0
				**% of time in motion**	**0.1**	**0.013**	**3.51 (−30.94; 22.81)**	**6.14 (2.11; 13.34)**	**0**	**+**
**Hawaii**	*Sicyopterus stimpsoni*	41	Fish length (cm)	**Bout duration (s)**	**0.13**	**0.022**	**11.28 (7.22; 20.22)**	**−1.46 (−3.62; −0.47)**	**0**	**-**
				**Net climbing speed (cm/s)**	**0.11**	**0.03**	**−0.17 (−1.02; 0.38)**	**0.17 (0.03; 0.37)**	**0**	**+**
				**Climbing only speed (cm/s)**	**0.24**	**0.001**	**−0.33 (−1.23; 0.17)**	**0.26 (0.14; 0.48)**	**0**	**+**
				% of time in motion	0.008	0.59	—	—	0	0
	*Sicydium punctatum*	54	Fish length (cm)	Bout duration (s)	0.009	0.5	—	—	0	0
				Net climbing speed (cm/s)	0.0005	0.87	—	—	0	0
**Dominica**				**Climbing only speed (cm/s)**	**0.13**	**0.008**	**1.39 (1.08; 1.80)**	**−0.15 (−0.28; −0.05)**	**0**	**-**
				**% of time in motion**	**0.2**	**<0.001**	**8.56 (−3.5; 16.33)**	**5.2 (2.72; 9.05)**	**0**	**+**
	*Cotylopus acutipinnis*	26	Fish length (cm)	Bout duration (s)	0.01	0.56	—	—	0	0
				**Net climbing speed (cm/s)**	**0.18**	**0.03**	**0.009 (−0.17; 0.12)**	**0.04 (0.01; 0.1)**	**0**	**+**
				**Climbing only speed (cm/s)**	**0.39**	**0.001**	**0.31 (−0.19; 0.62)**	**0.23 (0.13; 0.39)**	**0**	**+**
				% of time in motion	0.01	0.62	—	—	0	0
**La Réunion**	*Sicyopterus lagocephalus*	103	Fish length (cm)	**Bout duration (s)**	**0.05**	**0.024**	**−1.54 (−21.47; 1.25)**	**1.29 (0.62; 6.12)**	**0**	**+**
				Net climbing speed (cm/s)	0.014	0.23	—	—	0	0
				**Climbing only speed (cm/s)**	**0.08**	**0.004**	**0.47 (0.33; 0.61)**	**0.05 (0.018; 0.088)**	**0**	**+**
				% of time in motion	0.016	0.2	—	—	0	0

The suction produced by the pelvic disk was comparable in Hawaiian and Dominican adult gobies, but was substantially lower for species from Réunion, with mean suction in *S. lagocephalus* 36% less than that from *L. concolor* and *S. punctatum*, and pelvic suction pressures of *C. acutipinnis* less than half of the values from the Hawaiian and Dominican species (Table 1). As Réunion species exhibited poorer suction performance, these effects seem likely to be unrelated to climbing style (Table 1). However, adult *C. acutipinnes* exhibited the strongest allometric trend of pelvic suction generation with body size (Table 3). All other species, except *S. lagocephalus*, also displayed positive allometry of pelvic sucker performance with respect to growth in size (Table 3). This relationship was also present for the oral sucker of *S. stimpsoni*; however, *S. lagocephalus* did not exhibit any discernible relationship between body mass and suction performance for either the pelvic or oral sucker (Table 3). Despite these differences observed between the waterfall climbing species and climbing styles, climbing kinematics are similar for the three powerburst climbing species (Fig. 2), and the two inching species (Figs. 2, 3), respectively.

**Table 3:** Slope and intercept coefficients from RMA regressions (± 95% interval) of pelvic (all species) and oral (*Sicyopterus* spp. only) suction performance on body mass for adults of waterfall climbing stream gobies from Hawai’i, Dominica, and Réunion. Data for Hawaiian and Dominican species are derived from previously published studies (Maie et al. 2012). ΔPps, pelvic suction pressure differential; BM, body mass. Scaling pattern is indicated as isometric (0) or positively allometric (+) compared to the expected slope of 0 for isometry (Maie et al. 2012).

Island	Species	Suction structure	N	X	Y	r²	*P*-value	Intercept (95% confidence interval)	Slope (95% confidence interval)	Expected RMA by isometry*	Allometry
	*Lentipes concolor*	pelvic	9	log (BM)	log (ΔPps)	0.82	0.001	0.029 (-0.141; 0.144)	**0.522 (0.333; 0.801)**	0	+
	*Sicyopterus stimpsoni*	pelvic	15	log (BM)	log (ΔPps)	0.85	<0.001	−0.026 (−0.141; 0.060)	**0.585 (0.457; 0.756)**	0	+
**Hawaii**		mouth	15	log (BM)	log (Δpos)	0.67	<0.001	−0.045 (−0.165; 0.029)	**0.313 (0.203; 0.491)**	0	+
**Dominica**											
	*Sicydium punctatum*	pelvic	12	log (BM)	log (ΔPps)	0.80	<0.001	0.031 (−0.101; 0.126)	**0.411 (0.286; 0.584)**	0	+
	*Cotylopus acutipinnis*	pelvic	19	log (BM)	log (ΔPps)	0.88	<0.001	−0.146 (−0.229; −0.079)	**0.961 (0.796; 1.166)**	0	+
	*Sicyopterus lagocephalus*	pelvic	13	log (BM)	log (ΔPps)	0.06	0.43	–	–	0	0
**La Réunion**		mouth	13	log (BM)	log (Δpos)	0.05	0.47	–	–	0	0

## Discussion

This study tests the hypothesis that more recent functional innovations are less diverse in their performance across species than earlier-evolving innovations (e.g., Wainwright and Price 2016), and that patterns of diversity among juveniles are retained among adults (e.g., Blob et al. 2008b). In juveniles of climbing goby species, the more recently evolved inching climbing style superficially appeared to exhibit lower functional diversity than powerburst climbing; however, inching species achieved similar performance through different ratios of motion and rest, exhibiting a “masked” or hidden functional variation that complicated interpretations of relative diversity across climbing styles (Blob et al. 2019). Our new data on adult climbing gobies show a different pattern from our data on juveniles. Kruskal-Wallis tests indicate that body-length normalized net climbing speeds (including rest) differ between the two inching species compared (*S. stimpsoni* and *S. lagocephalus*), but do not differ significantly across the three powerburst species compared (*L.concolor, S.punctatum*, and *C. acutipinnis*) (Table 1). In fact, it is one of the inching species (*S. stimpsoni* from Hawai’i) that stands out with faster net climbing performance (0.13 BL/s) than the other taxa (all < 0.07 BL/s). Although adults of most climbing species show similar net performance, they each achieve this performance through different, species-specific combinations of speed during movement and portion of time spent moving (Table 1). For example, the powerburst species that shows the fastest movement while climbing, *C. acutipinnis*, spends the least time moving, yielding net performance similar to that of other powerburst species. These results show that, as in juveniles, similar levels of functional performance can be produced through multiple pathways, illustrating a broader phenomenon (Blob et al. 2019) within which the frequently recognized pattern of “many-to-one mapping of structure to function” is a specific example (Alfaro et al. 2005; Wainwright et al. 2005).

Inching species (*S. stimpsoni* and *S. lagocephalus*) also show species-specific combinations of speed during movement and the portion of time spent moving. Still, for these taxa, such patterns produce markedly different net performance. *Sicyopterus stimpsoni* exhibits a faster net speed than *S. lagocephalus* due to both moving more quickly and spending significantly more time moving (Table 1). These differences were not driven by the larger mean size of *S. lagocephalus*, as a re-analysis of those *S. lagocephalus* overlapping in size with *S. stimpsoni* shows that bout duration for the reduced *S. lagocephalus* cohort (n = 33) was virtually unchanged, while % time in motion only increased by 2.3% (to 47.3%) and was still well short of the 67% time in motion measured for *S. stimpsoni.* Given that net climbing speed is similar between *S. lagocephalus* and three outgroup species of powerburst climbers, it appears that the performance levels of adult *S. stimpsoni* represent a derived condition within the sicydiine lineage. Nonetheless, the limited kinematic differences within each climbing style are striking (Figs. 2, 3), a pattern also observed among juveniles of these goby species (Blob et al. 2019). In this context, performance differences within climbing styles are likely related to other, non-kinematic aspects of locomotor function. One possibility is differences in the proportions of slow versus fast fiber types in the axial muscles, which have been found to show functionally correlated differences between powerburst and inching species from Hawai’i (Cediel et al. 2008) and within subpopulations of *S. stimpsoni* (Blob et al. 2020). Fiber-type data are not available for *S. lagocephalus*, but could help determine the extent to which differences in this trait contribute to variation in goby climbing performance.

Intraspecific variability in performance and kinematic data were apparent in both powerburst and inching climbers among adults and juveniles (Table 1, Fligner-Kileen analysis). While intraspecific variability differed between species for most performance and kinematic variables, differences in the magnitude of that variability were not driven by climbing styles: in both adults and juveniles, powerburst and inching species commonly grouped together in post-hoc pairwise comparisons of homogeneity of variance. However, comparing results from Fligner-Killeen analyses between juveniles and adults (Table 1), which species grouped together for each variable frequently shifted between these age groups, suggesting that patterns of performance emerging during early life history may not substantially constrain outcomes for adults.

We cannot rule out that differences in the method of specimen collection influenced the performance we observed, as Hawaiian and Dominican gobies were collected exclusively with dip nets, whereas Réunion gobies were collected exclusively via electroshocking. Both collection methods can introduce biases (for example, biases towards less mobile fish when captured with a dipnet; lingering effects of electroshocking in subsequent experiments). However, the common acclimation period following capture and the consistency of performance within each group and across various experimental years provide indirect evidence that capture methods were inconsequential in our subsequent analyses of locomotor performance. Moreover, prior studies of the effects of electroshocking suggest that gross behavioral changes are limited to the first few hours after capture and that normal behaviors can be expected within 24 hours (Mesa and Schreck 1989), which coincides with the acclimation interval used in the current study.

Another aspect of performance that might contribute to locomotor differences across species is the adhesive strength of their suckers (Maie et al. 2012; Maie et al. 2013; Taft et al. 2017; Maie and Blob 2021; Palecek et al. 2021; Palecek et al. 2022; Maie et al. 2025; Palecek-McClung et al. 2025). The positive allometry in suction generated by pelvic disks that we observed in adults from four of our study species as they increased in size (Table 2) is consistent with observations that adults of all five species retain the ability to climb waterfalls despite increases in body mass as they grow through adulthood (Blob et al. 2007; Schoenfuss et al. 2011; Lagarde et al. 2018). The steepest slope and, therefore, the greatest allometric increases in pelvic suction performance were observed in *C. acutipinnis* (Table 2). This pattern might contribute to this species having the highest size-normalized climbing-only speed among adults of the species we compared (Table 1), despite having the lowest climbing-only speed among juveniles of powerburst species (Blob et al. 2019). Moreover, among inching species, the faster-climbing *S. stimpsoni* exhibited high suction performance and positive allometry (Tables 1, 2).

In contrast, the slower *S. lagocephalus* showed the lowest suction performance across the species we compared (Table 1) and no significant size-correlated pattern in suction pressure change (Table 2). Due to the size limitations of our equipment, it was not possible to collect comparable pressure data from smaller juvenile individuals. However, results from our sample suggest that variation in sucker structure and function may be another source of variation contributing to comparative patterns in climbing performance across climbing gobies (Maie et al. 2013; Palecek et al. 2022; Palecek-McClung et al. 2025).

When comparing climbing performance within species between juveniles (Schoenfuss et al. 2011; Blob et al. 2019) and adults (this study), no clear patterns emerged that were consistent across climbing styles. Similarly, overlapping regression slopes for several variables in both inching species, for instance, would have suggested that the more recently evolved behavior was less variable. Conversely, similar overlapping regression slopes across powerburst species could be interpreted to indicate that variability emerged with the evolution of the novel inching behavior. However, results from our scaling analyses were quite mixed. For example, net climbing speed increased with positive allometry in three of the five species, with two powerburst and one inching species climbing relatively faster as adults. Bout duration became longer with size in one inching species and declined in the other, while % time in motion only changed in one powerburst species (Table 3). Adult inching climbers in the current study were 2–3x as large as their previously studied juvenile conspecifics (Schoenfuss et al. 2011; Blob et al. 2019). However, body-size-adjusted climbing performance (net speed, including rest) remained similar between adult and juvenile *S. stimpsoni* (0.13 BL/s vs. 0.10 BL/s), with performance achieved in part by an increase in time spent in motion among adults (67% vs. 54%). In contrast, *S. lagocephalus* adults spent less time in motion than their juvenile conspecifics (45% vs. 47%), the only studied species (powerburst or inching) to do so. Overall, these patterns provide little evidence that the performance variability noted across juvenile climbing goby taxa (Blob et al. 2019) is sustained through growth to adult size.

While all waterfall climbing goby species migrate into new habitats (McDowall 2004) and increase in length roughly to the same degree during growth to adulthood, a greater range of net climbing performance was observed in the two inching species. In these species, ontogenetic growth is preceded by a dramatic reorganization of the body during metamorphosis (Schoenfuss 1997; Schoenfuss et al. 1997). Once in adult habitats, sexual selection may further constrain or expand functional diversity by either aligning with or acting counter to natural selection (Johansson et al. 2009). For taxa that experience such circumstances, expectations for the diversity of functional performance through ontogeny can be complicated to forecast. Mapping expected patterns of functional diversity (based on the above-stated hypothesis) on measured performance parameters for juvenile and adult gobies (Table 4, for juvenile data, see Schoenfuss et al. 2011; Blob et al. 2019), it is apparent that performance diversity is as common in the older functional innovation of powerburst climbing as it is in the more recent locomotor innovation of inching. The multitude of outcomes highlights the complexity of factors that can influence the trajectory of locomotor function across ontogeny (Carrier 1996; Herrel and Gibb 2006). Beyond the relative ages of functional innovations, data on additional factors such as changes in diet, habitat, and sexual selection may provide critical context for forecasting and interpreting performance outcomes across ontogeny. In addition, there are approximately 120–150 species of amphidromous sicydiine gobies, and our limited selection of species and genera may not reflect the full range of innovations associated with waterfall climbing locomotion. Although no reports are available to our knowledge, other climbing styles may exist or climbing styles may be further modified to accommodate species of greater size or in differing environmental settings. The current study highlights the need to cast an even wider geographic and phylogenetic net to fully assess the range of functional innovations related to waterfall climbing in these taxa.

**Table 4:** Summary of differences (“diverse”) and similarities (“similar”) in performance parameters related to the climbing ability of adults among the five species of Sicydiine gobies. Juvenile data from Schoenfuss et al. (2011) and Blob et al. (2019). *nm* = not measured.

	Juveniles		Adults	
	*Powerburst*	*Inching up*	*Powerburst*	*Inching up*
**Max fin angle**	Diverse	*nm*	Diverse	Similar
**Bout duration**	Diverse	Diverse	Similar	Similar
**Net climbing speed [BL/s]**	Diverse	Similar	Similar	Diverse
**Climbing only speed [BL/s]**	Similar	Diverse	Diverse	Similar
**% time in motion**	Similar	Similar	Diverse	Diverse
**Pelvic suction**	*nm*	*nm*	Diverse	Diverse

## Supplementary Material

obag044_Supplemental_Files

## Data Availability

In addition to data presented in the Supplementary File, data will be made available upon request.
