# Supplementary material for: Ontogenetic Change in Performance: Do Innovations Constrain Performance in Gobiid Fishes?
Source: Integr Org Biol. 2026 Aug 4;8(1):obag044. doi: 10.1093/iob/obag044 (PMC13494557; doi:10.1093/iob/obag044)
Supplement: Supplementary file 1 [file obag044_supplemental_files.zip › Alternate Language Abstracts_FINAL 016_Schoenfuss.docx]

**Abstract – Spanish**

Las innovaciones recientes en una especie pueden ampliar las oportunidades para acceder a nuevos recursos o a hábitats previamente inaccesibles. Con el tiempo, el comportamiento novedoso puede diversificarse en respuesta a un paisaje adaptativo o canalizarse, debido a presiones selectivas convergentes, hacia un intervalo más restringido de desempeño funcional. Las presiones selectivas también cambian a lo largo de las distintas etapas del desarrollo, lo que puede favorecer aún más la diversificación o limitar el desempeño. Para poner a prueba estas hipótesis alternativas, comparamos los cambios ontogenéticos en el desempeño de un comportamiento locomotor novedoso —el ascenso de cascadas— en cinco especies anfídromas de peces góbidos de los océanos Pacífico, Caribe e Índico. Dos especies ascienden las cascadas mediante un movimiento de avance por tramos (inching), una innovación funcional derivada que alterna los movimientos de las ventosas pélvica y oral. Las especies restantes ascienden las cascadas mediante breves «impulsos de potencia» (powerbursts) de natación, seguidos de largos períodos de descanso durante los cuales la ventosa pélvica permanece adherida al sustrato de la cascada. En los adultos, la velocidad neta de ascenso (incluidos los períodos de descanso) fue similar entre las tres especies que emplean powerbursts y una de las especies que utiliza inching; sin embargo, en uno de los taxones que emplean inching se observó una velocidad derivada mayor, lo que sugiere una mayor diversidad funcional en la innovación evolutiva más reciente. No obstante, los datos cinemáticos indican que niveles similares de desempeño pueden alcanzarse mediante múltiples vías funcionales. Además, los patrones de diversidad funcional observados entre especies durante la etapa juvenil no se mantienen de manera consistente en la adultez, lo que pone de manifiesto la diversidad de factores que pueden influir en la trayectoria de los cambios ontogenéticos de la función.

**Abstract – German**

Jüngere evolutionäre Innovationen innerhalb einer Art können die Möglichkeiten erweitern, neue Ressourcen oder zuvor unzugängliche Lebensräume zu erschließen. Im Laufe der Zeit kann sich ein neuartiges Verhalten als Reaktion auf eine adaptive Landschaft diversifizieren oder durch konvergierende Selektionsdrücke auf ein engeres Spektrum funktioneller Leistungsfähigkeit kanalisiert werden. Selektionsdrücke verändern sich zudem im Verlauf der Ontogenese, wodurch die Diversifizierung weiter gefördert oder die Leistungsfähigkeit eingeschränkt werden kann. Zur Prüfung dieser alternativen Hypothesen verglichen wir ontogenetische Veränderungen der Leistungsfähigkeit eines neuartigen Fortbewegungsverhaltens – des Wasserfallkletterns – bei fünf amphidromen Grundelarten aus dem Pazifik, der Karibik und dem Indischen Ozean. Zwei Arten erklimmen Wasserfälle mittels einer Inching-Bewegung, einer abgeleiteten funktionellen Innovation, bei der die Bewegungen der Bauch- und Mundsaugscheibe abwechselnd eingesetzt werden. Die übrigen Arten erklimmen Wasserfälle mithilfe kurzer Powerbursts des Schwimmens, auf die lange Ruhephasen folgen, während der die Bauchsaugscheibe am Substrat des Wasserfalls haftet. Bei adulten Tieren war die Netto-Klettergeschwindigkeit (einschließlich der Ruhephasen) bei den drei Powerburst-Arten und einer Inching-Art ähnlich. Bei einem Inching-Taxon wurde jedoch eine abgeleitete, höhere Klettergeschwindigkeit festgestellt, was auf eine größere funktionelle Vielfalt der jüngeren Innovation hindeutet. Kinematische Daten zeigen jedoch, dass vergleichbare Leistungsniveaus über mehrere funktionelle Mechanismen erreicht werden können. Darüber hinaus setzen sich die bei juvenilen Tieren beobachteten Muster funktioneller Vielfalt zwischen den Arten nicht in eindeutiger Weise bis ins Erwachsenenalter fort. Dies unterstreicht die Vielfalt potenzieller Einflüsse, die den Verlauf ontogenetischer Veränderungen der Funktion prägen können.

**Abstract – French**

Au sein d'une espèce, des innovations récentes peuvent élargir les possibilités d'accéder à de nouvelles ressources et/ou à des habitats auparavant inaccessibles. Ce nouveau comportement peut se diversifier, au fur et à mesure du temps, en réponse au paysage adaptatif, ou au contraire être canalisé par des pressions de sélection convergentes vers une diversité de performance plus restreinte. Les pressions de sélection évoluent également au cours des différents stades de la vie, ce qui peut soit favoriser la diversification soit limiter les performances. Afin de tester ces deux hypothèses alternatives, nous avons comparé les changements de performances au cours de l’ontogénie d'un comportement locomoteur novateur, la grimpe des cascades, chez cinq espèces de gobies amphidromes des océans Pacifique, Atlantique (Caraïbes) et Indien. Deux espèces grimpent les cascades grâce au mode de franchissement « *inching »*, une innovation fonctionnelle dérivée qui alterne les mouvements des ventouses pelvienne et orale. Les autres espèces grimpent les cascades grâce au mode de franchissement « *powerbursts* », qui alterne de courtes périodes de déplacements suivies de longues périodes de repos durant lesquelles les individus se maintiennent au substrat grâce à leur ventouse pelvienne. La vitesse nette d'ascension (temps de repos inclus) était similaire chez les adultes des trois espèces « *powerbursts »* et chez une des espèces « *inching »*. En revanche, une vitesse plus élevée a été observée chez un autre taxon « *inching »*, ce qui suggère une plus grande diversité fonctionnelle au sein de cette innovation récente. Toutefois, les données de cinématique montrent que des niveaux de performance similaires peuvent être atteints par plusieurs voies fonctionnelles. De plus, les patrons de diversité fonctionnelle observés chez les juvénile ne se retrouvent pas systématiquement chez les adultes, ce qui souligne la diversité des facteurs susceptibles d'influencer les changements fonctionnels au cours de l'ontogénie.

**Abstract – Korean**

한 종(種) 내에서 생겨나는 혁신은 새로운 자원이나 서식지를 차지할 수 있는 기회를 만들어 낼 수 있다. 시간이 흐름에 따라, 새로이 진화된 행동은 적응지형도(adaptive landscape)에 의해 다양화되거나, 수렴적인 선택압(convergent selective pressure)에 의해 발현 범위가 제약될 수 있다(narrowed performance window). 선택압은 또한 생애주기에 따라 변하는데, 이에 의해 다양화가 더욱 촉진되거나 능력(performance)이 제약될 수 있다. 이러한 대립가설들을 검증해보고자, 우리는 태평양, 카리브해, 그리고 인도양의 양측회유성(amphidromous) 망둥어(gobiid fish) 다섯 종들의 새로운 이동행동인 폭포등반의 성장과정 중 변화를 비교했다. 다섯 종 중 두 종은 애벌레처럼 꿈틀거리며(inching) 폭포를 오르는데, 이는 골반과 입의 흡반(sucker)을 번갈아 사용하는 파생된(derived) 기능적 혁신이다. 두 종을 제외한 나머지 종들은 짧은 powerburst(순간적인 폭발적 움직임)와, 그 직후 골반 흡착판을 이용해 폭포 벽(substrate)에 잠시 붙어있는 휴식기를 통해 폭포를 오른다. 성어(成魚)중에서는 (휴식기를 포함한) 총 등반 속도가 3개의 powerburst 종과 1개의 inching 종 사이에 비슷했지만, 나머지 하나의 inching 망둥어들 중에서는 더 빠른, 파생된 등반 속도가 관찰되었다. 이는 비교적 최근의 진화적 혁신에는 더 많은 기능적 다양성이 있음을 암시한다. 그렇지만, 운동학적 데이터는 비슷한 수준의 운동능력이 여러 개의 기능적 경로(functional pathway)를 통해 달성될 수 있다고 보여준다. 더욱이, 미성어(未成魚)때 여러 종에서 관찰되는 기능(function)의 다양성 분포는 성어로 온전하게 보존되지 않기 때문에, 이는 기능의 성장과정 중 변화(ontogenetic change)에 영향을 미칠 수 있는 잠재 원인의 넓은 범위를 보여준다.

**Abstract**

新行为在种群中的诞生有时意味着获取新资源和栖息地的可能性。随着时间推移，这些新的行为或因适应性地形而变得更加多样化，或被趋同的选择压力压缩在狭窄的表现区间内。而选择压力本身又可能会随着生长阶段发生变化，这既可能促使功能进一步分化，也可能反过来限制行为表型的范围。为了检验这些假设，本研究对比了来自太平洋、加勒比海和印度洋五种虾虎鱼类的瀑布攀爬表现—一种新颖的位移运动—在个体发育过程中的变化。其中两种虾虎鱼采用“蠕动式”攀爬，即交替使用腹鳍吸盘和口部吸盘附着前进，这属于一种派生性的功能创新。其余三种则通过短促的“爆发式游动”攀爬，随后进入较长的休息期，期间依靠腹鳍吸盘附着在瀑布基质上。在成鱼中，三种爆发式游动物种与一种蠕动式物种具有相似的净攀爬速度（含休息时间）；但另一种蠕动式攀爬物种则表现出更快的攀爬速度，表明这一较晚出现的创新行为展现了更高的功能多样性。不过，运动学数据显示，相似的表现水平可以通过多种功能途径实现。此外，在幼鱼阶段观察到的种间功能多样性并不能一致地延续到成鱼阶段，这凸显了可能塑造个体功能变化轨迹的多种潜在影响。
